# Assessment of Knowledge and Practice on Hepatitis B Infection Prevention and Associated Factors among Health Science Students in Woldia University, Northeast Ethiopia

**DOI:** 10.1155/2020/9421964

**Published:** 2020-04-09

**Authors:** Teshome Gebremeskel, Tirfe Beshah, Mulugeta Tesfaye, Biruk Beletew, Ayelign Mengesha, Addisu Getie

**Affiliations:** ^**1**^ Department of Anatomy, College of Health Sciences, Woldia University, P.O. Box 400, Woldia, Ethiopia; ^2^Department of Nursing, College of Health Sciences, Woldia University, P.O. Box 400, Woldia, Ethiopia

## Abstract

**Background:**

Hepatitis B virus is a global problem, with 66% of all the world population living in areas where there are high levels of infection. HBV is the leading risk factor for HCC globally and accounts for at least 50% of cases of HCC. Medical and health science students, being part of the health-care system, are exposed to the infection as a risk as other health-care workers when they come in contact with patients and contaminated instruments.

**Objective:**

The main aim of this study was to assess the knowledge and practice of hepatitis B virus infection prevention and its associated factors among health science students in Woldia University.

**Methods:**

Institutional-based cross-sectional study was conducted from January 30 to May 30, 2019, among health science students of Woldia University who had previous clinical attachments. Two hundred students were selected by the systematic random sampling method. Association of dependent and independent variables was computed using a bivariable and multivariable logistic regression model. P<0.05 was considered as significantly associated.

**Result:**

The study revealed that, out of 200 participants, 96 (48%) have poor knowledge, whereas 104 (52%) showed good knowledge about HBV. Regarding the practice of participants, 79 (39.5%) of the students have good practice to prevent HBV, whereas 121 (59.5%) had poor practice towards HBV infection prevention.

**Conclusion:**

Based on the current study, greater than half of the students who participated in the study have good knowledge of hepatitis B infection prevention and most of the students have poor practice about infection prevention of hepatitis B virus.

## 1. Introduction

Hepatitis is an inflammation of the liver mainly caused by the hepatitis B virus. Hepatitis B virus is a global problem, with 66% of all the world population living in areas where there are high levels of infection. Hepatitis B virus is a very important public health problem affecting almost 10% of the world population [1]. The viral hepatitis pandemic takes a heavy charge on lives, communities, and health systems. It is responsible for an estimated 1.4 million deaths per year from acute infection and hepatitis-related liver cancer and cirrhosis—a toll comparable to that of HIV and tuberculosis. Of those deaths, approximately 47% are attributable to hepatitis B virus. Worldwide, approximately 240 million people have chronic hepatitis B virus infection and 130–150 million have chronic hepatitis C virus infection. Without an expanded and accelerated response, the number of people living with the hepatitis B virus is projected to remain at the current, high levels for the next 40–50 years, with a cumulative 20 million deaths occurring between 2015 and 2030 [2].

HBV is contagious and easy to be transmitted from one infected individual to another by blood contact, mother to child, unprotected sexual intercourse, sharing of eating utensils, and other barbershop and beauty salon equipment [3]. Hepatitis B is mainly transmitted through prenatal infection, skin, and mucous membrane infections caused by contaminated blood or body, sexual contacts, and injection drug abusers. Also, tattooing, ear piercing, acupuncture, and dialysis are procedures that increase the risk of infection. HBV is the leading risk factor for HCC globally and accounts for at least 50% of cases of HCC. Chronic liver disease due to hepatitis B virus (HBV) accounts for the majority of HCC cases and thus highly amenable to preventive measures [4, 5]. Hepatitis B infection mainly affects the liver that interrupts the liver's ability to perform vital functions and cause both acute and chronic diseases. Hepatitis virus has an insidious effect elsewhere in the body or has an extrahepatic effect. The prevalence of hepatitis B virus infection is higher in those persons exposed to blood products [6, 7].

Hepatitis B infection is common due to the gap in the sterilization technique of instruments or due to the improper hospital waste management as 10% to 20% health-care waste is regarded hazardous and it may create a variety of health risks. Among the health-care personnel, HBV is transmitted by the prick of infected, contaminated needles and syringes in the skin, or through accidental inoculation of the minute quantities of blood during surgical and dental procedures. Knowledge regarding the hepatitis B virus and safety precautions is needed to minimize the health-care settings acquired infections among health personnel [8]. The overall number of health-care workers per annum exposed to sharp injuries contaminated with HBV was estimated to reach 2.1 million [9, 10]. It can reduce the transmission of viral hepatitis to both users of health-care services as well as health-care workers through the rigorous application of universal precautions for all invasive medical interventions, consistent implementation of infection control practices, including safe injection measures in health-care and community settings. But it still occurs because of the absence or poor quality of screening in blood transfusion services [2].

Medical and health science students, being part of the health-care delivery system, are exposed to the infection as a risk as other health-care workers when they come in contact with patients and contaminated instruments. They are the first level of contact between patients and medical care. They are expected to undertake activities related to patient care at the beginning of their clinical years. Since medical and health science students are at increased risk of acquiring needle stick injury, and exposed to blood and blood products in their professional practice, conducting a study regarding the knowledge and practice level of students is very crucial for intervention to reduce the transmission of the virus.

This study assessed the knowledge and practice of students towards HBV infection prevention among health students in Woldia University. The result of this study will assist the stakeholders to be aware of the knowledge and practice level of students to prevent the burden of HBV infection.

## 2. Methods

Institutional-based cross-sectional study design was employed in Woldia University from January 30 to May 30, 2019. Woldia University is one of the public universities in Ethiopia, located north of Dessie and south east of Lalibela in Amhara region, and it is about 521 km from Addis Ababa. Currently, the University has two campuses, namely, Jenetober and Mersa College of agriculture. The Health Science College is found at Jenetober campus and has four departments which are Public Health, Nursing, Midwifery, and Medical Laboratory Science with 797 regular students under the college.

### 2.1. Source Population

All regular health science students in Woldia University.

### 2.2. Study Population

Regular health science students who had previous clinical exposure.

### 2.3. Sample Size Determination

The sample size for this study was determined by using the single population proportion formula by considering the following assumptions: The proportion of knowledge and practice on hepatitis B infection prevention was taken from the result of a previous similar study conducted among Wollo University Medical College students; from this study, the student who had good knowledge and good practice about hepatitis B infection prevention was 81.1% and 59.8%, respectively. By taking the prevalence which gives the highest sample value population proportion, 59.8% (*p*=0.598), level of significance 5% (*α* = 0.05), *Z*_*α*/2_ = 1.96, and margin of error 5% (*d* = 0.05). Finally, a 10% nonresponse rate was added, and then, the total sample size required for this study appeared to be 200 [11].

### 2.4. Sampling Technique

The study participants were selected from each department by proportion based on the total number of students in each department. The study participants were selected from each department among 3^rd^ (*n* = 22) and 4^th^ (*n* = 23) year Public Health and 3^rd^ (*n* = 19) and 4^th^ (*n* = 27) year Nursing students, then 2^nd^ (*n* = 18), 3^rd^ (*n* = 20), and 4^th^ (*n* = 31) year Midwifery, and 2^nd^ (*n* = 25) and 3^rd^ (*n* = 15) year Medical Laboratory students of Woldia University. A systematic random sampling method was used to select specific students from the classroom by using their checklist. Then the first student was selected by lottery method and the next student was selected in the interval according to their order in the list. In the case of absent students, the next student was taken as a respondent and the interval continue as in the previous.

### 2.5. Data Collection Method and Procedure

Data were collected by using a structured questionnaire and pretest before starting the actual data collection was conducted. The questionnaire was containing written consents, socio-demographic variables, knowledge, and practice questions towards hepatitis B infection prevention which were developed by adapting from different peer-reviewed literature. Then, after getting permission letter from Woldia University College of Health Science Department of Nursing, the participants were selected from each department student checklist by the systematic random sampling method.

### 2.6. Eligibility Criteria

Regular health science students who had previous clinical exposure were included in this study, whereas critically ill students at the time of data collection were excluded from the study.

### 2.7. Operational Definitions

  Good knowledge: Study participants who answer more than half of the knowledge questions correctly.
  Poor knowledge: Study participants who answer less or equal to half of the knowledge questions correctly.
  Good practice: Study participants who were able to answer more than half of practice item questions correctly.
  Poor practice: Participants who were unable to answer less or equal to half of the practice item questions correctly [12].

### 2.8. Data Quality Control

For ensuring data quality, training was given for data collectors and supervisors. After this, pretest of the questionnaire was conducted on 5% of students in Wollo University Health Science students a week before the actual survey, and necessary modification was done according to the gap identified. Data collection from the participant was carried out in their class room. The data collection process was strictly followed day to day by the supervisor and principal investigators and the collected data was checked its completeness and consistency every day by the supervisor and principal investigators.

### 2.9. Data Processing and Analysis

Data were coded and entered into Epi-Data version 4.2.0 and exported to SPSS Version 20 for analysis. Exploratory data analysis was done to check missing values. Descriptive frequencies were calculated to describe the study population concerning relevant variables and presented by tables. Binary logistic regression analysis was conducted to assess the crude association between dependent and independent variables. Then, variables which show association in binary logistic regression analysis and have a *P*-value less than 0.25 were entered into the multivariable logistic regression model to control the possible effect of confounding. Finally, significant factors were identified based on AOR include in 95% confidence level and a *P*-value less than 0.05.

### 2.10. Ethical Consideration

Ethical clearance was obtained from Woldia University College of Health Science, Research and Community Service Office. Permission letter was obtained from Woldia University College of Health Science, Department of Nursing. The purpose and objective of the study were explained to the respondents. Then, verbal consent was taken from each participant after clearly explaining the purpose of the study. They were informed to withdraw from the study at any time and/or to refrain from responding to questions if they were not interested to participate by any reason. Finally, data were collected in the way in which we were not inflicted right, culture, norms, and ethical issues of participants.

## 3. Results

A total of 200 students from four different departments were participated in the study making a response rate of 100%. The majority of the participants (85%) were with in the age group of 20–24, and 115 (57.5%) of the respondents were males. The majority (182 (91%)) of the study participants were single in marital status, and more than half (56%) of the respondents came from urban areas.

### 3.1. Knowledge of Hepatitis B Transmission and Prevention

Out of the 200 participants, 96 (48%) have poor knowledge, whereas 104 (52%) showed good knowledge about HBV. Thirty-two percent of 65 (32.5%) of the study participants did not know about hepatitis B postexposure prophylaxis (Table 1).

### 3.2. Practices of Respondents on Hepatitis B Infection Prevention

Out of 200 participants, 79 (39.5%) of students have good practice, whereas 121 (59.5%) had poor practice towards HBV infection prevention. 149 (74.5%) of the respondents were never screened for HBV, and 16 (8%) of the students were vaccinated against HBV. Among the vaccinated group, 2 (12.5%) completed all 3 doses of the vaccination schedule, and the remaining 4 (25.4%) and 10 (62.5%) students took only first and second dose, respectively (Table 2).

### 3.3. Factors Associated with Knowledge and Practice of Students

In this study, academic year and field of study of the respondents were significantly associated with knowledge of participants. Being 4^th^ and 3^rd^ year are 14 and 8 times more knowledgeable about HBV than being 2^nd^-year student, respectively (*P* < 0.001). An infield of study, being nurse is less likely knowledgeable towards infection prevention and transmission of HBV than medical laboratory students (AOR = 0.071, *P* < 0.001). Being a health officer and midwifery student also less likely knowledgeable towards infection prevention of HBV than medical laboratory students (AOR = 0.130 and 0.129, respectively) (Table 3).

In the current study, 137 (68.5%) of the respondents reported that the reason for not vaccinated against the hepatitis B virus is due to lack of resources; also 21 (10.5%) of the respondents reported lack of resource is the reason for not using personal protective equipment. Likewise 86 (43%) of respondents respond the reason for not screening for the hepatitis B virus is due to lack of resources followed by 43 (21.5%) feeling of low-risk status [Table 4].

## 4. Discussion

In the current study, 52% of participants had good knowledge of hepatitis B virus infection prevention. This result was in line with the study conducted in Pakistan in which the knowledge level reported among medical students of seven medical colleges of Karachi was 57.1%, a study conducted in Saudi Arabia in which 50% of medical students in the University of Dammam, the study conducted among medical students of Tanta University, Egypt, which was 57.85%, and a study conducted in Haramaya University, 56.5% of students were knowledgeable regarding hepatitis B virus infection prevention [13–16]. But the current finding was lower than the study conducted among students at Cacodyl University, Ivory Coast, revealed that 69.4% of the students were knowledgeable [17], the study conducted among medical students of the University of Yaoundé I, Cameroon, reported that 83.2% [18], and the study conducted at Wollo University Medical College students revealed 81.1% have good knowledge on hepatitis B infection prevention [11]. The higher knowledge in other studies may be due to most of the study participants were in-service students from different health-care settings compared to fresh regular students in this study.

In the current study, academic year and field of study were significantly associated with knowledge of participants. Being 4^th^ and 3^rd^ year (AOR = 14.00 and 8.00, respectively, *P* < 0.001) are more knowledgeable than 2^nd^-year students. This may be due to, as year of study increases, increasing in practical experience and theoretical knowledge by the students. Regarding field of study, being a nurse is less likely knowledgeable towards infection prevention towards HBV than medical laboratory students (AOR = 0.071, *P* < 0.001). Being health officer and midwifery (AOR = 0.130 and 0.129) also less likely knowledgeable towards infection prevention of HBV than medical laboratory students, respectively. This may be due to medical laboratory students have better exposure to infection prevention during different sample collection procedures for their day to day activity. This study is in line with the study conducted at Haramaya University, which is a statistically significant association between the field of study and knowledge of HBV was observed among medical science students, but the year of study was not significantly associated with knowledge of the students [15]. A similar study conducted at Gondar University among health science students revealed the field of study was significantly associated with knowledge of HBV infection prevention [19].

This study revealed that out of 200 participants, 39% of them have a good practice on hepatitis B infection prevention. This finding was lower compared to the study conducted on Wollo University medical college students, which showed that 50.0% of students have a good practice on hepatitis B infection prevention, and the study conducted among medical students of Tanta University, Egypt, in which the students who have good practice were 68.1% [11, 16]. This may be due to the difference in resource allocation, knowledge, and perception of students in the area.

In the current study, lack of resource/resource limitation was the main reason for not screening for hepatitis B virus (43%) and not vaccinated for hepatitis B virus (68.5%). This study was comparable with similar study done at Sudan; unawareness of vaccine, expensiveness of the vaccine, fear or perceived high risk of HBV infection through occupational exposures, fear of contracting hepatitis from the vaccine, doubt of the efficacy of the vaccine, and absence postexposure prophylaxis were the reason of not being vaccinated [20].

## 5. Conclusion

Based on the current study, greater than half of the students have good knowledge of hepatitis B infection prevention; however, most of the students have poor practice about infection prevention on HBV. Study department and field of studies are factors for knowledge of the students towards infection prevention of HBV, whereas lack of resource, low-risk status, and doubt of vaccine efficacy were the main reason not to be screened and vaccinated for hepatitis B virus.

## 6. Recommendations

Woldia University College of Health Science should give special emphasis on HBV infection prevention training for health science students before they start their clinical attachment.

It is also recommended that the university should allocate resources for complete vaccination of all health science students before they start clinical attachment despite their profession.

## Figures and Tables

**Table 1:** Knowledge of participants on hepatitis B virus infection prevention among health science students in Woldia University, Northeast Ethiopia, 2019.

Variables	Yes	No
Hepatitis B can transmit by fecal-oral?	67 (33.5%)	133 (66.5%)
Hepatitis B can transmit by mother to child?	178 (89%)	22 (11%)
Hepatitis B can transmit by contaminated water?	43 (21.5%)	157 (78.5%)
Hepatitis B can transmit by sexual contact?	179 (89.5%)	21 (10.5%)
Carriers can transmit hepatitis B?	175 (87.5%)	25 (12.5%)
Hepatitis B can transmit by handshaking?	143 (71.5%)	57 (28.5%)
Hepatitis B can transmit by contact with open wounds?	174 (87%)	26 (13%)
Hepatitis B can transmit by blood and body fluid?	189 (94.5%)	11 (5.5%)
Hepatitis B can transmit by unsterilized syringe, the needle?	184 (92%)	16 (8%)
Do you know about the vaccine of hepatitis B?	150 (75%)	50 (25%)
The vaccine can prevent HBV?	178 (89%)	22 (11%)
Is there a laboratory test for HBV?	183 (91.5%)	17 (8.5%)
Is there postexposure prophylaxis for HBV?	135 (67.5%)	65 (32.5%)
Does HBV cause liver cancer?	174 (87%)	26 (13%)
HBV is treatable?	153 (76.5%)	47 (23.5%)

**Table 2:** Practice of participants on hepatitis B infection prevention among health science students in Woldia University, Northeast Ethiopia, 2019.

Variables	Yes	No
Always wear glove during giving care?	177 (88.5%)	23 (11.5%)
To dispose of the needle properly?	176 (88%)	24 (12%)
Have you ever screened?	48 (24%)	152 (76%)
Have you been vaccinated for hepatitis B?	16 (8%)	184 (92%)
Do you ask the screening of blood before transfusion?	146 (73%)	54 (27%)
Did you ask a new syringe before use?	172 (86%)	28 (14%)
Have you participated in health education?	93 (46.5%)	107 (53.5%)

**Table 3:** Bivariable and multivariable results of computed variables affecting knowledge of participants regarding HBV among health science students in Woldia University, 2019.

Variables	Knowledge				
	Poor	Good	COR	AOR	CI (95%)
Age in years					
15–19	2	2			
20–24	79	91	1.297	1.396	0.185–10.560
25–29	14	11	0.786	0.747	0.087–6.442
≥30	1	0	0.000	0.000	

Sex					
Male	46	58	0.984	0.345	0.0123–0.962
Female	39	46			

Residence					
Urban	40	48	0.33	1.54	0.788–3.039
Rural	56	56			

Field of study					
Nursing	26	20	0.492	0.071	0.019–0.258
Health officer	21	24	0.731	0.131	0.037–0.458
Midwifery	33	25	0.891	0.269	0.084–0.854
Medical laboratory	16	35			

Academic year					
Second-year	29	14			
Third-year	35	40	2.367	8.0	**2.57-24.8** ^*∗*^
Fourth-year	32	50	3.327	14.0	**4.17-44.0** ^*∗*^

AOR = adjusted odds ratio; COR= crude odds ratio. ^*∗*^Significantly associated.

**Table 4:** Results of factors affecting the practice of respondents on hepatitis B infection prevention among health science students in Woldia University, 2019.

Factors	Frequency
Not using PPE	
Lack of resource	21 (10.5%)
Negligence	1 (0.5%)

Reason for not screened	
Lack of resource	86 (43%)
Low-risk status	43 (21.5%)
Lack of knowledge	23 (11.5%)

Reason for not vaccinated	
Busy schedule	4 (2%)
Low-risk status	33 (16.5%)
Lack of resource	137 (68.5%)
Doubt of efficacy	15 (7.5%)

## Data Availability

The datasets used and/or analyzed during the current study are available from the corresponding author upon reasonable request.
